# Impact of a Modified Version of Baby-Led Weaning on Dietary Variety and Food Preferences in Infants

**DOI:** 10.3390/nu10081092

**Published:** 2018-08-15

**Authors:** Brittany J. Morison, Anne-Louise M. Heath, Jillian J. Haszard, Karen Hein, Elizabeth A. Fleming, Lisa Daniels, Elizabeth W. Erickson, Louise J. Fangupo, Benjamin J. Wheeler, Barry J. Taylor, Rachael W. Taylor

**Affiliations:** 1 Department of Human Nutrition, University of Otago, Dunedin 9054, New Zealand; brittany.morison@gmail.com (B.J.M.); anne-louise.heath@otago.ac.nz (A.-L.M.H.); jill.haszard@otago.ac.nz (J.J.H.); liz.fleming@otago.ac.nz (E.A.F.); lisa.daniels@otago.ac.nz (L.D.); liz.erickson@otago.ac.nz (E.W.E.); louise.fangupo@otago.ac.nz (L.J.F.); 2 Department of Food Science, University of Otago, Dunedin 9054, New Zealand; karen.hein@otago.ac.nz; 3 Department of Women’s and Children’s Health, University of Otago, Dunedin 9054, New Zealand; ben.wheeler@otago.ac.nz; 4 Office of the Dean, Dunedin School of Medicine, University of Otago, Dunedin 9054, New Zealand; barry.taylor@otago.ac.nz; 5 Department of Medicine, University of Otago, Dunedin 9054, New Zealand

**Keywords:** complementary feeding, infant, food variety, food preferences, baby-led weaning

## Abstract

The aim of this study was to determine whether food variety and perceived food preferences differ in infants following baby-led instead of traditional spoon-feeding approaches to introducing solids. A total of 206 women (41.3% primiparous) were recruited in late pregnancy from a single maternity hospital (response rate 23.4%) and randomized to Control (*n* = 101) or BLISS (*n* = 105) groups. All participants received government-funded Well Child care. BLISS participants also received support to exclusively breastfeed to 6 months and three educational sessions on BLISS (Baby-Led Weaning, modified to reduce the risk of iron deficiency, growth faltering, and choking) at 5.5, 7, and 9 months. Food variety was calculated from three-day weighed diet records at 7, 12, and 24 months. Questionnaires assessed infant preference for different tastes and textures at 12 months, and for ‘vegetables’, ‘fruit’, ‘meat and fish’, or ‘desserts’ at 24 months. At 24 months, 50.5% of participants provided diet record data, and 78.2% provided food preference data. BLISS participants had greater variety in ‘core’ (difference in counts over three days, 95% CI: 1.3, 0.4 to 2.2), ‘non-core’ (0.6, 0.2 to 0.9), and ‘meat and other protein’ (1.3, 0.8 to 1.9) foods at 7 months, and in ‘fruit and vegetable’ foods at 24 months (2, 0.4 to 3.6). The only differences in perceived food preferences observed were very small (i.e., <5% difference in score, at 12 months only). Infants following the modified Baby-Led Weaning were exposed to more varied and textured foods from an early age, but only an increased variety in ‘fruit and vegetable’ intake was apparent by two years of age.

## 1. Introduction

Parents have traditionally introduced their infant to complementary foods by spoon-feeding them puréed foods. As the infant learns to manage solid foods, a wider variety of foods with increasingly complex textures are offered [1]. Although the progression may include some finger foods, these do not usually represent a significant component of the diet until later in complementary feeding [2,3,4,5,6]. This parent-led approach to complementary feeding differs substantially from Baby-Led Weaning (BLW), which advocates bypassing purées and instead recommends that the infant self-feeds all their foods in a solid form from the start of weaning [7,8,9,10]. Advocates of BLW suggest this approach leads to healthier food preferences due to the infant being exposed to greater food variety and role modeling through ‘joining in’ at the family meal [11,12,13].

No research appears to have investigated whether baby-led approaches influence food variety, even though different first foods are typically offered [14,15,16,17]. Learning to like ‘healthy’ foods is modifiable [18,19], and infants who are repeatedly exposed to foods varying in taste and texture are more willing to eat those foods and other novel foods later in childhood [20,21]. Although advocates claim that BLW infants prefer a wider range of foods [7], this has only been examined in one small study, where significant differences were seen for just one of eight food groups examined [22].

We recently completed a randomized clinical trial (RCT) determining whether growth differed in infants following baby-led versus parent-led approaches to complementary feeding (Baby-Led Introduction to SolidS [BLISS] study) [23]. This analysis of preplanned secondary outcomes focuses on potential differences in food variety and preferences from 7 to 24 months of age.

## 2. Materials and Methods

BLISS compared modified BLW with usual care (traditional spoon-feeding; Control). BLISS followed the general principles of BLW (infants feed themselves handheld foods and are involved in family meal times) but was modified in response to concerns that BLW may increase the risk of iron deficiency, choking, and growth faltering [24]. As protocol [25] and primary outcome [23] papers have been published, only relevant information is provided here. The study was approved by the Lower South Regional Ethics Committee (LRS/11/09/037), and written informed consent was obtained from all adult participants before randomization.

**Figure 1:**
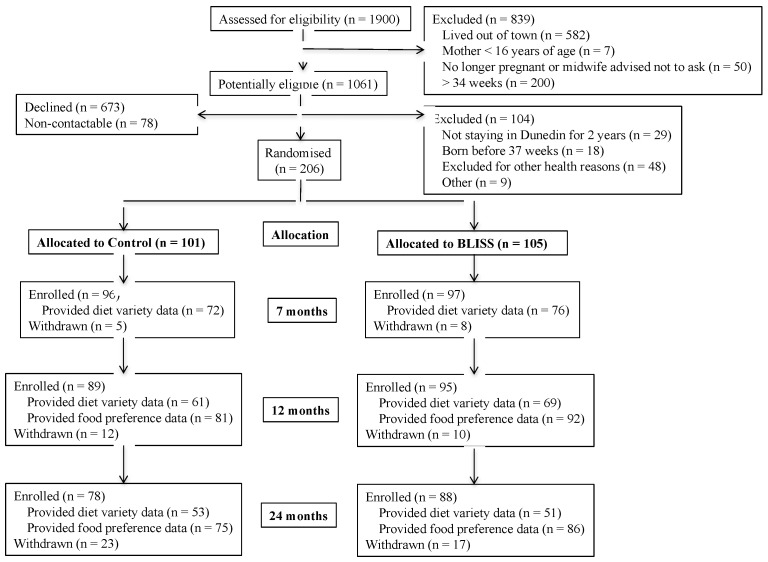
Flow of participants through the study.

Women were recruited in late pregnancy from sequential bookings (December 2012–March 2014) at the only maternity hospital in Dunedin, New Zealand (>97% of all births). Exclusion criteria were applied in late pregnancy (not living locally, mother ≤16 years, booked after 34 weeks gestation) and after birth (prematurity, congenital abnormality likely to affect feeding/growth), producing a final sample size of 206 participants (Figure 1). The participants were randomly allocated to Control (*n* = 101) or BLISS (*n* = 105) groups, using random length blocks after stratification for parity (first child, subsequent child) and maternal education (non-tertiary, tertiary) by the study biostatistician.

Both control and intervention children received Well Child care, a nationally funded health program from antenatal until 5 years of age [26,27]. Families in the BLISS group received eight additional contacts. Five contacts (antenatal to 5 months of age) were delivered by an International Board Certified Lactation Consultant (IBCLC) to assist prolonging exclusive breastfeeding and delaying the introduction of complementary foods until 6 months of age. A trained BLISS researcher met individually with each family at 5.5, 7, and 9 months of age to provide advice and support about the BLISS approach to complementary feeding. The essential characteristics are: 1) offer foods that the infant can pick up and feed themselves, 2) offer one high-iron food at every meal, 3) offer one high-energy food at each meal, and 4) offer foods prepared in a way that is suitable for the infant’s developmental age to reduce the risk of choking and avoid offering foods listed as high-choking-risk foods [25]. A variety of pretested [28] resources provided ideas for adapting family meals following a baby-led philosophy, while promoting responsive feeding practices. Parents were encouraged to offer a variety of healthy foods to their infants, wherever possible offering foods from the family meal.

Demographic information was obtained at baseline (late pregnancy) from hospital records (birth weight, infant sex, parity, level of household deprivation [29]) and questionnaire (maternal education, employment status, ethnicity, self-reported pre-pregnancy height and weight), after the mothers had provided consent but before randomization to intervention group.

Three-day weighed diet records were collected on randomly assigned non-consecutive days (one weekend day, two week days) over a three-week period at 7, 12, and 24 months of age to assess food variety. When relevant, the parents were asked to record the weight of infant formula powder, added water, and total prepared weight of the formula offered. Estimated total daily volumes were used for breast milk (750 g/day at 7 months, 448 g/day at 12 months [30], 59 g/breastfeed at 24 month [31]), with the reported volume of prepared formula subtracted from these amounts as appropriate for mixed-fed infants. The participants received written and oral instructions for completing the records [25].

Food variety scores were calculated for four food groups (‘core foods’, ‘non-core foods’, ‘meat and other protein’, and ‘fruit and vegetables’) on the basis of the method of Scott et al [32]. Diet records were only included if all three days had been completed. Individual foods were assigned to food groups (Table 1). Each individual food was only counted once over the three days, regardless of how many times that particular food had been offered. Foods that were similar (e.g., different cultivars of apples) were only counted once, but different forms of the food were counted as individual items (e.g., raw apple and stewed apple would provide two counts). Mixed food dishes and commercial baby foods were broken down into their component parts (e.g., a steak and cheese pie would provide three counts: ‘meat and other protein’ (steak), ‘non-core’ foods (pastry), and ‘core’ foods (cheese)). However, the mixed dish or baby food itself only provided one point for the total variety score. For each food or beverage item, the parents also indicated whether the item was puréed, mashed, diced, naturally smooth, whole, or liquid (Table 2).

Food preferences at 12 months of age were assessed by asking the parents to indicate whether their toddler had ever been offered (never offered, 1–3 times, 4–6 times, 7–10 times, 11 or more times, don’t know) and subsequently consumed (‘yes, they always eat it’, ‘yes, they sometimes eat it’, ‘yes, but they rarely eat it’, ‘no, they reject it after tasting’, and ‘no, they refuse to taste it’), each of 21 foods. The 21 foods were chosen from those most commonly consumed by toddlers in New Zealand [33] and assigned to six taste categories (‘sweet’, ‘salty’, ‘savory-meat’, ‘savory-non-meat high-protein’, ‘savory-vegetable’, and ‘savory-French fries’) and four texture categories (‘smooth’, ‘lumpy’, ‘chewy’, ‘crunchy’). This taste and texture allocation was decided after asking a separate group of 14 parents with age-appropriate children to assign each food to what they considered was the most appropriate category, with final classification established where there was at least 50% agreement. Twenty of the 21 foods were consistently assigned to a certain taste category; the only food not assigned was peanut butter, for which 43% of parents indicated a savory taste and 36% a salty taste. Parents were in agreement regarding the texture of 16 of these foods, with five foods not consistently assigned to a specific texture category (olives, tomato, baloney, cheese, French fries). Thus, 20 foods were included in analyses for taste, and 16 foods for texture.

At 24 months of age, food preference was assessed using an existing questionnaire that included single foods (e.g., banana), groups of similar foods (e.g., savory snacks including potato chips and crackers), and mixed dishes (e.g., lasagna) [34]. Minor modifications were made to the questionnaire to make it appropriate for New Zealand families (e.g., ‘salad greens’ was replaced with ‘lettuce’).

The mothers indicated how much the child liked each of these foods using one of six response options: ‘dislikes a lot’, ‘dislikes a little’, ‘neither dislikes nor likes’, ‘likes a little’, ‘likes a lot’, and ‘hasn’t tried it’. Because of the relatively small size of our sample, we used the factors of Wardle et al. [34] to create preference scores for ‘vegetables’ (*n* = 9 items), ‘fruit’ (*n* = 9 items), ‘meat and fish’ (*n* = 8 items), and ‘desserts’ (*n* = 6 items). The scores were obtained as the mean liking on the response scale from 1 (dislikes a lot) to 5 (likes a lot) of all items within each factor. The missing items were imputed with the mean of the remaining items in the scale.

### Statistics

The primary aim of our RCT was to determine whether BLISS improves the body mass index (BMI) at 12 months of age. Sample size calculations were based on detecting a difference in BMI of 0.4. Sample size calculations were not undertaken in relation to dietary variety or food preferences, as these were secondary outcomes of the main trial (i.e., part of our dietary quality measures) [25]. Differences between BLISS and Control groups are therefore presented with 95% CI for interpretation without *P*-values, after adjustment for maternal education and parity (stratification variables) and infant sex. Statistical analysis was carried out using Stata 14.2 (Stata Corporation, College Station, TX, USA). The demographic differences between participants who provided dietary data at 12 or 24 months and those who did not were compared using t-tests for continuous variables and chi-squared tests for categorical variables.

Linear regression was used to determine the mean difference in food variety scores (counts) between BLISS and Control groups at 7, 12, and 24 months and the mean difference in exposure and preference scores for different tastes and textures at 12 months. Residuals were plotted and visually assessed for homogeneity of variance and normality.

Due to skewed data, group medians (25th, 75th percentiles) are presented for mean daily proportions of forms in which food was consumed at 7, 12, and 24 months and for food preference scores at 24 months using the factors of Wardle et al. [34]. Differences between groups were assessed using bootstrapped median regression with 100 replications.

## 3. Results

### 3.1. Participants

Control and BLISS participants were similar at baseline (Table 3). Almost half of all mothers were university-educated (48.5%), and fewer participants came from households with higher levels of deprivation (21.4%) than would be expected nationally (30%). Participant retention was high (Figure 1): 166 participants (80.6%) remained at two years of age. While virtually all participants provided food preference data (94%–97%), fully completed three-day diet records were provided by a lower percentage of participants (76.7% at 7 months, 70.7% at 12 months, 62.7% at 24 months). Families who provided any data for analyses at 12 months (*n* = 174) did not differ from those who did not provide data (*n* = 32) in terms of maternal BMI (*p* = 0.338), maternal education (*p* = 0.211), household deprivation (*p* = 0.795), or group (*p* = 0.203), although participating mothers were older than non-participants (31.9 versus 27.8 years, *p* < 0.001).

As reported previously [23], BLISS infants were exclusively breastfed for longer than Control infants, and more BLISS infants met the World Health Organization guideline to delay solids to 6 months than Control infants. However, there were no significant group differences in the estimated intake of breast milk (all *p* ≥ 0.940) or infant formula (all *p* ≥ 0.170) at any time point, nor in the proportion of infants who were introduced to solids earlier than 4 months (5.2% in BLISS versus 12.8% in Controls, *p* = 0.057). BLISS children consumed a greater proportion of their food as ‘whole’ foods at each age, particularly at 7 months (31% of foods compared with 8% in Controls, *p* < 0.001, Table 2).

### 3.2. Food Variety

At 7 months of age, BLISS participants had greater total food variety (difference, 95% CI: 3.0, 1.1 to 4.8, Table 4), consisting of greater variety in the intake of ‘core foods’ (1.3, 0.4 to 2.2), ‘non-core foods’ (0.6, 0.2 to 0.9), and ‘meat and other protein’ (1.3, 0.8 to 1.9), with no difference in the ‘fruit and vegetable’ variety (−1.1, −2.4 to 0.2). By 24 months of age, the only significant difference between groups was a higher ‘fruit and vegetable’ variety count in BLISS compared with Control children (2.0, 0.4 to 3.6).

### 3.3. Food Preferences

At 12 months of age, food preferences were assessed for different tastes (Table 5) and textures (Table 6). Children in both groups had been offered each taste 4.8 (‘salty’) to 7.4 (‘savory-meat’ and ‘savory-non-meat high-protein’) times on average by 12 months (Table 3). Although BLISS participants were offered ‘savory-vegetables’ (difference in number of times, 95% CI: 0.8, 0.01 to 1.5) and ‘savory-non-meat high-protein foods’ (1.3, 0.6 to 2.1) more often than Control participants, a difference in perceived preference (i.e. foods actually consumed) was only apparent for ‘savory-non-meat high-protein’ foods and was small (0.2, 0.04 to 0.4; i.e., 4.7%, 0.9% to 9.3% of Control value). Similarly, in terms of texture, BLISS infants were more likely to be offered (1.1, 0.4 to 1.7) lumpy foods but only slightly more likely to consume them (0.2, 0.01 to 0.3; i.e., 4.8%, 0.2% to 7.1% of Control value) (Table 6). At 24 months of age (Table 7), overall preference scores were high for ‘fruit’ and moderately high for ‘meat and fish’ and ‘desserts’, with slightly lower values for ‘vegetables’. However, no significant differences in these preference scores were apparent between groups at this age.

Because group differences in the duration of exclusive breastfeeding and timing of the introduction of complementary foods could explain some of the observed differences in food exposures and preferences, post-hoc mediation analyses [35] were run for all statistically significant results, with duration of exclusive breastfeeding or age of introduction of complementary foods as mediators. Neither variable was a mediator for any of the relationships found. In fact, the adjustment for age of introduction of complementary foods strengthened the differences seen between the groups for variety of ‘core foods’ and ‘non-core foods’ and amount of liquids at 7 months of age; amount of whole food and preferences for ‘savory-non-meat high-protein’ and lumpy foods at 12 months of age; amount of whole food and liquids at 24 months, although all differences were small and did not influence the interpretation of the findings reported above.

## 4. Discussion

Our data demonstrate that a baby-led approach to complementary feeding leads to increased dietary variety and greater exposure to more textured foods at a young age. Infants following BLISS had greater variety in the intake of ‘core foods’, ‘non-core foods’, and ‘meat and other protein sources’ at 7 months and in the ‘fruit and vegetable’ variety at 24 months. By 12 months of age, they also had increased preference for ‘savory-non-meat high-protein’ foods and were more likely to be offered, and consume, lumpy foods. However, these differences were very small and appeared to be transitory, in that no differences in preference for ‘vegetables’, ‘fruit’, ‘meat and fish’, or ‘desserts’ (or the foods classified as ‘savory-non-meat high-protein’ and ‘lumpy’ in the analysis at 12 months) were apparent by 24 months of age.

No previous studies appear to have examined whether the dietary variety differs in infants following baby-led rather than parent-led approaches to complementary feeding, despite the expectation that opportunities to eat a more adult-type diet with the family would lead to a wider variety of foods being consumed [11,12,13]. There are several potential reasons why the dietary variety might be increased with a baby-led approach to feeding. Breastfeeding exposes infants to a wider variety of tastes than occurs with formula feeding [18,36], and BLISS infants were exclusively breastfed for several weeks longer than Control infants [23]. In addition, others [37] have shown that the early introduction of complementary foods may be detrimental to food variety; young children who had been introduced to complementary foods before 6 months of age were 2.5 times more likely (95% CI: 1.1–5.7) to consume a limited variety of foods than those who had been introduced to solids after 6 months. In our sample, BLISS infants were on average introduced to complementary foods several weeks later than Control infants, and a significantly greater proportion did not introduce solids until 6 months of age or later (64.6% of BLISS infants compared with 18.1% of Control infants) [23]. However, mediation analysis showed that the differences in food variety between the groups were not explained by either duration of exclusive breastfeeding or age when complementary feeding started. It is likely, therefore, that it was the baby-led approach to complementary feeding itself that was responsible for the greater dietary variety in the BLISS infants at 7 months. Group differences in dietary variety at 7 months of age were no longer apparent by 12 months. Interestingly, although no statistically significant differences in the ‘fruit and vegetable’ variety were observed in infancy, BLISS children consumed a wider variety of fruit and vegetables at two years of age, suggesting some benefit to dietary variety over the longer-term. It would be useful to determine whether this increased variety continues to older ages, given the health benefits of consuming a wide variety of fruit and vegetables [38].

As with food variety, limited research has examined food preferences in baby-led compared to parent-fed infants. Townsend et al. [22] reported that of eight different food groups examined, differences in preference were only apparent for one: baby-led infants were more likely to prefer ‘carbohydrate’ foods, despite lower exposure to these foods than spoon-fed infants. Repeated exposure to a food is one of the primary determinants of its acceptance [20], and the child must be allowed to experience the flavor and taste of the fruit or vegetable to learn to like it [39], perhaps as many as ten times [40]. Our data demonstrate that BLISS infants were offered vegetables more often than Control infants at 12 months of age, but that this was not reflected in greater consumption. However, our infants were only exposed to the vegetables we investigated around six times on average, which might explain this lack of effect. Texture is also important. Letting young children experience more varied textures is associated with fewer food refusals [41] and a better acceptance of weaning foods later on [42]. However, although children in the BLISS study were offered lumpy foods more often and showed a greater preference for these foods at 12 months of age, a preference for the same foods was not apparent at 24 months of age, suggesting little effect on long-term healthy food preferences. These findings are consistent with the observation that food fussiness was significantly lower in BLISS infants than Control infants at 12 months but not at 24 months [23].

The strengths of our study include the randomized trial design, the high level of adherence to the intervention, and the repeated measurement of dietary variety and food preferences using a range of tools. The use of a randomized trial design overcomes the known differences in demographics and parental feeding practices that exist in those who choose to follow BLW rather than traditional feeding practices [14,43], and that could conceivably influence the development of food preferences. Infants who had been randomized to the BLISS intervention group were significantly and substantially more likely to follow a baby-led approach to infant feeding than infants in the Control group [23], so we can be confident that the null findings are not due to poor adherence to the intervention. We also determined whether group differences in breastfeeding or the age of solids introduction might have influenced our findings, given their known effect on variety and development of food preferences in young children [36]. However, adjustment for these variables had little effect on the group differences observed.

Our study also has some limitations. This was an analysis of secondary outcomes of interest from a wider RCT. Although study retention was 81% overall at two years of age, only 78–84% of parents completed the questionnaires on food preferences, and 50–72% provided three complete days of weighed diet record data for food variety assessment. However, no differences in a range of demographic variables (except maternal age) were observed between the families who did and those who did not provide data. Also, dietary variety was estimated at each time point using only three days of diet records, which may be insufficient, given that the accurate assessment of variety in children may need as much as two weeks [44]. However, our diet records were collected on non-consecutive days, which improves the accuracy of dietary variety estimations when compared with consecutive recording days [44]. At 12 months of age, we only assessed taste and texture preferences using a relatively limited range of foods. However, this food list was developed from a larger pool of foods, and a separate validation study was undertaken to ensure consistency in parental interpretation of the tastes and textures represented by the foods. Finally, our two groups differed in contact time, which could potentially affect the outcomes.

## 5. Conclusions

Our findings demonstrate that a baby-led approach to complementary feeding increases dietary variety and exposure to more textured foods at a young age. By two years of age, the only difference observed was a higher variety in ‘fruit and vegetable’ intake. In contrast, any impact on perceived food preferences appeared to be only transitory.

## Figures and Tables

**Table 1:** Foods included in each food variety grouping.

**Core foods (No Maximum Count)**
Dairy products
Milk alternative ^1^
Any cheese
Sweetened yoghurt ^2^
Unsweetened yoghurt ^2^
Custard
Low-fat dairy ^3^
High-fat dairy ^4^
Ice cream
Milk not as a drink ^5^
Grains
Individual breakfast cereals counted separately
Baby rice cereal
Breads-white
Breads-whole meal or wholegrain
Breads-novelty ^6^
Rice
Pasta
Crackers
Cereal bars
Baby rusks
Other grain products
Milk
Breast milk
Infant formula
Cow’s milk as a drink
**Non-core foods (no maximum count)**
Savoury
Pies
Burgers
Battered fish
Pizza
Fried chicken
Fried potatoes ^7^
Pastries ^8^
Dips ^9^
Savoury muffins or scones
Croissants
Potato chips
Popcorn
Corn chips
Other salty snacks
Other takeaway foods
Sweet
Cakes or slices
Muffins or fruit loaves
Sweet scones or pancakes
Sweet pastries
Biscuits ^10^
Individual ‘other desserts’ counted separately
Candy
Chocolate
Frozen ice block ^11^
Other drinks
Soda
Fruit-flavored drink
Flavored milk drink
Fruit juice
Tea
Milo, chocolate, and malt drink
Coffee
**Meat and other protein (maximum = 16)**
Eggs
Peanut butter
Nuts or seeds
Baked beans
Hummus
Legumes
Vegetarian meat substitutes
Beef
Lamb
Pork
Venison
Chicken or turkey
Fish or shellfish
Sausages
Processed meats or cold cuts ^12^
Offal and other unspecified meats
Pâté
**Fruit and vegetables (no maximum count)**
Individual fruits ^13^ and vegetables ^14^ counted separately

^1^ soy, almond, rice, bran, oat milks; ^2^ includes soy; ^3^ cottage cheese, low-fat cheeses; ^4^ cream, sour cream, cream cheese; ^5^ milk or milk substitutes were counted as dairy produce if they were added to cooking or cereals; ^6^ fruit, nut, seed, vegetable bread; ^7^ French fries, hash browns, fritters; ^8^ sausage rolls, savories; ^9^ excludes hummus; ^10^ includes semi-sweet biscuits; ^11^ includes sorbet; ^12^ includes bacon and ham; ^13^ includes avocado; ^14^ includes mushrooms, excludes French fries.

**Table 2:** Form of food ^1^ consumed by the participants at each time point (from three-day weighed diet records).

	7 Months			12 Months			24 Months		
	Control (*n* = 77)	BLISS (*n* = 85)	*p* ^8^	Control (*n* = 69)	BLISS (*n* = 75)	*p* ^8^	Control (*n* = 56)	BLISS (*n* = 57)	*p* ^8^
Puréed ^2^	8.8 (2.6, 18.5)	3.7 (0, 9.0)	0.009	0 (0, 3.6)	0 (0, 3.1)	>0.999	0 (0, 0)	0 (0, 0)	>0.999
Mashed ^3^	6.1 (1.3, 13.5)	3.1 (0, 7.5)	0.054	2.2 (0, 9.6)	0.8 (0, 3.1)	0.235	0 (0, 1.7)	0 (0, 1.4)	>0.999
Diced ^4^	0 (0, 2.2)	0 (0, 2.1)	>0.999	2.8 (0, 5.6)	0 (0, 5.1)	0.084	1.1 (0, 5.9)	1.1 (0, 6.3)	0.927
Smooth ^5^	3.7 (0, 8.6)	7.5 (3.7, 11.9)	0.209	5.2 (1.4, 9.7)	4.7 (1.7, 8.8)	0.422	8.6 (3.7, 16.0)	8.8 (4.2, 12.8)	0.543
Whole ^6^	7.9 (2.6, 21.1)	31.0 (20.5, 36.4)	<0.001	20.8 (13.2, 31.7)	28.2 (19.2, 34.6)	0.025	37.7 (29.6, 49.2)	49.7 (37.7, 62.5)	<0.001
Liquid ^7^	54.0 (43.8, 65.9)	44.9 (36.6, 55.3)	0.008	57.1 (47.4, 66.4)	57.4 (49.6, 66.6)	0.631	36.6 (23.0, 51.3)	32.8 (20.7, 42.4)	0.008

^1^ Data presented as median (25th, 75th percentile) of the percentage of daily food and drink intake by weight (excluding drinking water). ^2^ Food blended together to make a smooth consistency, ^3^ mashed by hand to make a lumpy consistency, ^4^ chopped into small pieces and needing a spoon to eat it, ^5^ originally smooth in texture with no modification, ^6^ pieces that are large enough to easily handle and eat by hand, ^7^ breast milk, infant formula, cow’s milk, juice; ^8^ median regression bootstrapped with 100 replications, adjusting for maternal education and parity and infant sex. Number of missing responses for form of food at: 7 months, 171/6709 food items (2.5%); 12 months, 28/6673 food items (0.4%); 24 months, 221/5362 (4.1%). These food items were included in the calculation of participant’s total weight of food intake but not assigned to a form of food. BLISS: Baby-Led Introduction to SolidS.

**Table 3:** Characteristics of the study population at baseline.

	Category	Control (*n* = 101)	BLISS (*n* = 105)
		*n* (%)	*n* (%)
**Maternal variables**			
Age (years) ^1^	Mean (SD)	31.3 (6.2)	31.3 (5.0)
Pre-pregnancy BMI ^2^	Mean (SD)	25.6 (5.6)	25.9 (6.3)
Education	School only	29 (28.7)	34 (32.4)
	Post-secondary	19 (18.8)	24 (22.9)
	University	53 (52.5)	47 (44.8)
Parity	First child	42 (41.6)	43 (41.0)
	Subsequent child	59 (59.4)	62 (59.0)
Ethnicity	NZEO	85 (84.2)	83 (79.0)
	Māori or Pacific	10 (9.9)	15 (14.1)
	Asian	6 (5.9)	7 (6.7)
**Household variables**			
Household deprivation ^3^	1–3 (Low)	29 (28.7)	31 (29.5)
	4–7	49 (48.5)	53 (50.5)
	8–10 (High)	23 (22.8)	21 (20.0)
**Infant variables**			
Birth weight (g) ^4^	Mean (SD)	3531 (486)	3509 (451)
Sex ^5^	Male	53 (52.5)	43 (41.0)
	Female	47 (47.5)	62 (59.0)

Data expressed as *n* (%) except where indicated; Data missing for 1 ^1^, 7 ^2^, 3 ^4^, and 1 ^5^ participants; NZEO refers to New Zealand European and Others; BMI: body mass index. ^3^ Uses the New Zealand Index of Deprivation 2013 which combines nine variables from the 2013 census relating to communication (no access to the internet at home), income (receiving a means tested benefit or living below income thresholds), unemployment, qualifications, home ownership, single parent families, living space, and transport access. A deprivation score is provided for each meshblock which is a geographical unit defined by Statistics New Zealand containing about 60–10 people. The score reflects the extent of material and social deprivation and is used to construct defiles from 1 (least deprived) to 10 (most deprived).

**Table 4:** Food variety (counts) at 7, 12, and 24 months of age over the three days of the three-day weighed diet record.

	7 months			12 months			24 months		
Food group ^1^	Control (*n* = 72)	BLISS (*n* = 76)	Difference ^2^ (95% CI)	Control (*n* = 61)	BLISS (*n* = 69)	Difference ^2^ (95% CI)	Control (*n* = 53)	BLISS (*n* = 51)	Difference ^2^ (95% CI)
Core foods	5.8 (2.9)	7.1 (2.4)	1.3 (0.4, 2.2)	9.4 (2.6)	9.3 (2.5)	0.0 (−0.9, 0.9)	9.8 (2.4)	9.7 (2.2)	0.1 (−0.7, 0.9)
Non-core foods	0.5 (1.0)	1.1 (1.1)	0.6 (0.2, 0.9)	2.3 (1.7)	2.2 (1.7)	−0.1 (−0.7, 0.5)	4.9 (2.7)	4.0 (2.6)	−0.7 (−1.8, 0.3)
Meat & other protein	1.9 (1.7)	3.2 (1.8)	1.3 (0.8, 1.9)	3.6 (1.6)	4.0 (1.7)	0.5 (−0.02, 1.1)	4.0 (1.6)	4.4 (1.8)	0.4 (−0.2, 1.1)
Fruit and vegetables	9.2 (4.1)	8.1 (3.7)	−1.1 (−2.4, 0.2)	11.9 (4.4)	11.1 (5.1)	−0.6 (−2.2, 1.1)	9.8 (4.2)	11.5 (4.1)	2.0 (0.4, 3.6)
Total food variety	13.1 (6.1)	15.9 (5.4)	3.0 (1.1, 4.8)	21.4 (5.8)	21.6 (6.0)	0.4 (−1.6 2.4)	24.6 (6.4)	25.3 (6.8)	1.3 (−1.1, 3.7)

Data presented as mean (SD). ^1^ Broad categories of foods were: ‘core foods’ (dairy, grains, milk), ‘non-core foods’ (savory and sweet snacks, drinks), ‘meat and other protein’, and ‘fruit and vegetables’. See Table 1 for food and drink items within each category; ^2^ Difference (95% CI) in variety counts in BLISS relative to Controls analyzed using linear regression, adjusting for maternal education and parity (stratification variables) and infant sex.

**Table 5:** Food exposure and perceived preference scores for different tastes at 12 months of age in relation to the complementary feeding style.

Taste category	Control (*n* = 81)	BLISS (*n* = 92)	Difference (95% CI) BLISS: Control ^3^
**Exposure score—i.e., offered to the infant ^1^**			
Sweet ^4^	6.0 (2.1)	5.7 (2.3)	−0.3 (−1.0, 0.4)
Savory-vegetable ^5^	5.9 (2.8)	6.5 (2.2)	0.8 (0.01, 1.5)
Savory-meat ^6^	7.1 (2.8)	7.7 (2.5)	0.4 (−0.3, 1.2)
Savory-non-meat high-protein ^7^	6.8 (2.7)	8.0 (2.5)	1.3 (0.6, 2.1)
Savory-French fries ^8^	6.8 (3.8)	7.0 (4.0)	0.2 (−1.0, 1.4)
Salty ^9^	5.0 (2.3)	4.6 (2.3)	−0.5 (−1.2, 0.2)
**Preference score—i.e., consumed by the infant ^2^**			
Sweet ^4^	4.5 (0.5)	4.5 (0.4)	0.0 (−0.1, 0.1)
Savory-vegetable ^5^	4.1 (0.8)	4.2 (0.6)	0.1 (−0.1, 0.3)
Savory-meat ^6^	4.4 (0.6)	4.5 (0.5)	0.1 (−0.1, 0.2)
Savory-non-meat high-protein ^7^	4.3 (0.7)	4.5 (0.5)	0.2 (0.04, 0.4)
Savory-French fries ^8^	4.7 (0.4)	4.6 (0.6)	−0.1 (−0.3, 0.1)
Salty ^9^	4.4 (0.8)	4.4 (0.8)	0.0 (−0.3, 0.3)

^1^ mean (SD) number of times this type of food was offered. Response options were: ‘never offered’, 1–’3 times’, ‘4–6 times’, ‘7–10 times’, ‘11 or more times’, and ‘don’t know’, and were coded as 0, 2, 5, 8.5, 11, and missing, respectively. For each food-type category, the mean number of exposures for the foods in that category was calculated for each participant. Missing values (or ‘don’t know’ responses) were excluded; ^2^ as data on intake were only available for foods that had been offered, preference score is presented as mean (SD) of the mean score of intake of the foods in that scale on a response scale from 1 (no, refuses to taste) to 5 (always eats when offered); ^3^ difference (95% CI) in scores in BLISS relative to Controls analyzed using linear regression, adjusting for maternal education and parity (stratification variables) and infant sex; ^4^ bananas, cookies, yogurt, raisins, breakfast cereals; ^5^ broccoli, cabbage, spinach, cauliflower, tomato; ^6^ baloney, ground meat, cooked meat cuts, sausage; ^7^ cheese, baked beans, egg; ^8^ French fries/hot chips/wedges; ^9^ yeast extract, olives.

**Table 6:** Food exposure and perceived preference scores for different textures at 12 months of age in relation to the complementary feeding style.

Texture category	Control (*n* = 81)	BLISS (*n* = 92)	Difference (95% CI) BLISS: Control ^3^
**Exposure score—i.e., offered to the infant ^1^**			
Smooth ^4^	8.0 (3.6)	7.6 (3.3)	−0.4 (−1.5, 0.6)
Lumpy ^5^	7.5 (2.3)	8.5 (1.9)	1.1 (0.4, 1.7)
Chewy ^6^	5.2 (2.3)	5.6 (2.1)	0.4 (−0.2, 1.0)
Crunchy ^7^	3.7 (2.7)	3.5 (2.6)	−0.2 (−1.0, 0.5)
**Preference score—i.e., consumed by the infant ^2^**			
Smooth ^4^	4.6 (0.6)	4.6 (0.6)	0.0 (−0.2, 0.2)
Lumpy ^5^	4.2 (0.5)	4.4 (0.4)	0.2 (0.01, 0.3)
Chewy ^6^	4.3 (0.5)	4.3 (0.5)	0.0 (−0.1, 0.2)
Crunchy ^7^	4.6 (0.5)	4.5 (0.5)	0.0 (−0.2, 0.1)

^1^ mean (SD) number of times this type of food was offered. Response options were: ‘never offered’, 1–’3 times’, ‘4–6 times’, ‘7–10 times’, ‘11 or more times’, and ‘don’t know’, and were coded as 0, 2, 5, 8.5, 11, and missing, respectively. For each texture category, the mean number of exposures for the foods in that category was calculated for each participant. Missing values (or ‘don’t know’ responses) were excluded; ^2^ as data on intake were only available for foods that had been offered, preference score is presented as mean (SD) of the mean score of intake of the foods in that scale on a response scale from 1 (no, refuses to taste) to 5 (always eats when offered); ^3^ difference (95% CI) in scores in BLISS relative to Controls analyzed using linear regression, adjusting for maternal education and parity (stratification variables) and infant sex; ^4^ yogurt, yeast extract; ^5^ ground meat, baked beans, egg, cauliflower, banana, broccoli; ^6^ raisins, spinach, cooked meat cuts, sausage, cabbage; ^7^ chunky peanut butter, breakfast cereals, cookies.

**Table 7:** Perceived food preference scores at 24 months of age in relation to the complementary feeding style [34].

Food Category	Control (*n* = 75)	BLISS (*n* = 86)	Difference (95% CI) BLISS: Control ^1^
‘Vegetables’ ^2^	3.7 (3.1, 4.1)	3.8 (3.2, 4.2)	0.1 (−0.2, 0.5)
‘Fruit’ ^3^	4.8 (4.4, 4.9)	4.8 (4.4, 4.9)	0.1 (−0.1, 0.2)
‘Meat and fish’ ^4^	4.2 (3.7, 4.6)	4.3 (4.0, 4.5)	0.1 (−0.2, 0.3)
‘Desserts’ ^5^	4.4 (3.8, 4.8)	4.4 (4.0, 4.8)	0.1 (−0.2, 0.4)

Data presented as median (25th, 75th percentile). Scores found as the mean liking (on a response scale from 1 (dislikes a lot) to 5 (likes a lot) for all items. Missing items were imputed with the mean of the remaining items in the scale; ^1^ difference (95% CI) in scores in BLISS relative to Controls analyzed using median regression, adjusting for maternal education and parity (stratification variables) and infant sex; ^2^ broccoli, cabbage, carrots, cauliflower, green beans, mushrooms, parsnips, lettuce, and tomato; ^3^ apples, bananas, oranges, grapes, peaches, strawberries, fruit juice, as well as ice cream, and frozen ice pops; ^4^ beef, lamb, pork, chicken, bacon, fried fish, fresh fish, and canned fish; ^5^ cream, cakes, sweet pastries, fruit-based desserts, custard, and dairy desserts.
